# Alexithymia and Symptoms of Post-Traumatic Stress Disorder: The Mediation Roles of Self-Compassion and Deficits in Emotion Regulation

**DOI:** 10.3390/ejihpe16020030

**Published:** 2026-02-18

**Authors:** George Fedorov, Glen Bates

**Affiliations:** Department of Psychological Sciences, School of Health Sciences, Swinburne University of Technology, Melbourne, VIC 3122, Australia; 102727204@student.swin.edu.auwi

**Keywords:** alexithymia, PTSD symptoms, self-compassion, emotional regulation, serial mediation, negative- and positive-emotion, vulnerability to mental disorder

## Abstract

Post-Traumatic Stress Disorder (PTSD) is a global mental health concern, with recent research focussing on the psychological mechanisms that contribute to its development and maintenance. Alexithymia, characterised by difficulty identifying and expressing emotions, has been identified as a potential risk factor for PTSD. This study was a preliminary investigation of a model of the relationship between alexithymia and PTSD symptoms, focussing on the potential mediating roles of self-compassion and difficulties in emotional regulation. Participants (*N* = 332), who were university students and members of the community, completed self-report measures of the key variables. As expected, alexithymia was strongly associated with higher levels of PTSD symptoms. Three mediation pathways were also significant. In one, alexithymia was associated with greater regulation difficulties for negative emotions, which was associated with higher levels of PTSD symptoms. In the second, higher alexithymia was associated with greater difficulties regulating positive emotions, which was associated with higher levels of PTSD symptoms. The final pathway involved a serial mediation in which higher alexithymia was associated with lower self-compassion, and lower self-compassion was associated with greater difficulties in regulating negative emotions, which were associated with higher PTSD symptoms. Contrary to expectation, self-compassion had no direct relationship with PTSD symptoms and did not relate to difficulties in regulating positive emotions. The general pattern of results was evident for the PTSD subtypes of negative alterations in cognitions and mood and alterations in arousal and reactivity. However, mediation by difficulties in regulating positive emotions was nonsignificant for the symptoms of re-experiencing and avoidance. The theoretical and clinical implications of the findings are discussed.

## 1. Introduction

Post-Traumatic Stress Disorder (PTSD) is a debilitating mental disorder that can develop after exposure to traumatic events, such as experiencing or witnessing threatened death, serious injury or violence (APA, 2022; Ozer & Weiss, 2004). Those affected by PTSD experience a range of symptoms, including intrusive re-experiencing of the trauma, avoidance behaviours, negative alterations in cognition and mood and alterations in arousal and reactivity. These symptoms impair their well-being and ability to function in daily life (Diagnostic and Statistics Manual-5 Text Revision [DSM-5-TR]; APA, 2022).

Not everyone who experiences a potentially traumatic event develops PTSD. In fact, only about 8% of individuals who experience a potentially traumatic event have ongoing symptoms of PTSD (Bistricky et al., 2017; Kilpatrick et al., 2013). Understanding the factors that contribute to the onset and maintenance of PTSD symptoms is crucial, therefore, for developing effective treatment and prevention strategies. In this study, we examined the relationship between alexithymia and the symptoms of PTSD. This was based on research that has established alexithymia as a vulnerability factor for mental disorders, including PTSD (Luminet & Nielson, 2025). We also considered difficulties in emotional regulation and the influence of a self-compassionate stance toward the self as potential mediators of this relationship. All these factors have strong associations with impaired emotional processing, which exacerbates trauma-related symptoms and hinders recovery (Fang et al., 2020; Kindred et al., 2024; Lilly & Valdez, 2012; Mehta et al., 2025).

## 2. The Alexithymia Construct and Symptoms of PTSD

### 2.1. Defining Alexithymia

Alexithymia is a multidimensional construct involving deficits in emotional engagement (Putica et al., 2021). Individuals with alexithymia struggle to become aware of and label their own emotions (*Difficulty Identifying Feelings*). This leaves them confused or unsure of what they feel. This difficulty extends to verbal expression (*Difficulty Describing Feelings*), limiting their capacity to communicate emotions to others and creating misunderstandings and a sense of isolation (Luminet & Nielson, 2025). Additionally, people with alexithymia tend to focus on external events or facts (*Externally Oriented Thinking*) rather than their internal emotional world. Instead, they adopt a more detached or pragmatic approach to emotions and constricted imaginal activity (*Paucity of Fantasy*) (Taylor et al., 2024).

The four components of the original model of alexithymia were derived from clinical observations of patients with psychosomatic complaints rather than from a theoretical framework. In the original model, the four components are believed to interact with one another. The focus of this model is on negative emotions given the anhedonic presentation of patients with severe alexithymia (Taylor et al., 2024). More recently, however, Preece and colleagues (Preece et al., 2018) have proposed an alternative theoretically based model of alexithymia. This model reconceptualises the relationships among the components of the construct and includes positive as well as negative emotions.

The attention–appraisal model of alexithymia is derived from Gross’s extended process model of emotion regulation (Gross, 2015). In this model, the difficulties associated with alexithymia are located within a four-part temporal framework. Awareness of an emotional state (situation phase) stimulates the direction of attention to the emotional response (attentional phase). An appraisal phase then follows in which the emotion being experienced is determined. This appraisal leads to a choice of responses directed at changing the emotion if needed (response phase). In this model, the externally oriented rather than emotion-focused-thinking component of alexithymia negatively influences the attention phase. In turn, difficulties identifying feelings and difficulties describing feelings disrupt the later appraisal phase and lead to the less effective use of emotion regulation strategies. Notably, the paucity-of-fantasy component is not included in the attention–appraisal model of alexithymia. This decision was based on recognition of the difficulties in measuring imaginal processes with self-report measures and the view that externally oriented thinking may also represent limited imaginal thinking (Preece et al., 2018; Taylor et al., 2024).

The inclusion of positive emotions alongside negative emotions in the attention–appraisal model reflects a broad conception of emotional processing. This includes the current recognition that positive as well as negative emotions are influenced by psychological distress (Weiss et al., 2020). Moreover, in Gross’s temporal model, the valence of the emotion is not fully determined by the processes of attention and appraisal, both of which are influenced by alexithymia. Thus, alexithymia is not restricted to negative emotions in the general population.

### 2.2. The Relationship Between Alexithymia and PTSD Symptoms

Alexithymia has a clear relationship with PTSD symptomatology (Putica et al., 2024). A meta-analysis by Edwards (2022) found that individuals with PTSD have more severe levels of alexithymic traits than the general population. Independent of diagnosis, higher levels of alexithymic traits are associated with elevated post-traumatic symptoms. This suggests that the relationship between alexithymia and PTSD symptoms is reciprocal. However, there is much evidence that alexithymia constitutes a relatively stable personality trait that is present to some degree in all people and independent of many psychological conditions (Luminet & Nielson, 2025). In a manner analogous to neuroticism and extraversion, relative differences in alexithymia remain the same over time while symptom measures change. For example, Larionow et al. (2025) found that alexithymia scores remained stable over seven months in a three-wave longitudinal study in a student sample. In contrast, measures of well-being, anxiety, depression and somatic concerns fluctuated over the three time points and did not predict alexithymia scores. Similar findings for clinical samples of people with depression, alcohol withdrawal and breast cancer confirm alexithymia as a risk factor (Luminet & Nielson, 2025).

Alexithymia is associated with each of the four core symptom types of PTSD. The first group of symptoms of re-experiencing trauma include flashbacks, intrusive memories and vivid recollections of traumatic events. These symptoms force individuals to confront trauma-related emotions. Alexithymia exacerbates these symptoms by interfering with attempts to process such experiences. Individuals high in alexithymia experience greater emotional dysregulation when faced with recurrent reminders of their trauma (Kooiman et al., 2002). Their inability to identify and communicate these intense emotions heightens distress during re-experiencing episodes. This is because emotions tied to the traumatic event resurface without the individual being able to manage them effectively (Litz et al., 2002). People with alexithymia also show a heightened sensitivity to trauma-related stimuli because they lack the emotional vocabulary and capacity for recognition needed to process these experiences. This worsens psychological impairment (Oglodek, 2022; Putica, 2024).

The second group of PTSD symptoms focusses on avoidance and involves efforts to evade trauma-related thoughts, feelings and reminders of the traumatic event. Avoidance intensifies efforts to suppress emotion expression in individuals with alexithymia (Putica et al., 2023). As people with alexithymia struggle to identify and articulate their emotional states, avoidance reinforces these difficulties, further impairing emotional awareness (Panayiotou et al., 2020). Hetzel-Riggin and Meads (2016) found that people with alexithymia engaged more often in avoidant coping mechanisms, which perpetuate the cycle of trauma-related emotional dysregulation. This suggests that the relationship between avoidance and alexithymia follows a feedback loop. The more an individual engages in avoidance, the less they can process their emotional experiences. This reinforces avoidance and the emotional suppression associated with alexithymia. Thus, avoidance blocks access to the emotional processing necessary for healing (Mehta et al., 2025).

Negative alterations in cognitions and mood is a third PTSD symptom type linked to alexithymia. These symptoms include persistent feelings of fear, horror, guilt, shame or anger. Alexithymia further intensifies such negative emotional states because difficulties in recognising and articulating emotions increases the sense of being overwhelmed by emotion (Fang et al., 2020). An inability to manage negative emotion or to feel positive emotions creates a feedback loop whereby unprocessed emotions contribute to greater psychological distress and more severe PTSD symptoms (Fang et al., 2020).

The final group of symptoms of PTSD, alterations in arousal and reactivity, involves heightened irritability, hypervigilance, concentration problems, insomnia and an exaggerated startle response. Research shows that individuals high in alexithymia have chronically elevated negative affect independent of external stressors (Kindred et al., 2024). In these individuals, there is a mismatch between heightened subjective emotional distress and autonomic arousal, suggesting dysregulated physiological–emotional responses (Connelly & Denney, 2007). Alexithymia amplifies physiological dysregulation, increasing difficulties in managing physical reactions to stressors (Putica et al., 2023). The inability to recognise and process emotions prolongs arousal states and perpetuates heightened physiological responses (Putica et al., 2023). Over time, this sustained hyperactivation contributes to chronic stress, sleep disturbances and increased psychological impairment, which impedes recovery (Fang et al., 2020).

## 3. Self-Compassion and Emotional Regulation as Mediators of the Relationship Between Alexithymia and Symptoms of PTSD

The extreme impairment caused by the symptoms of PTSD and alexithymia has encouraged investigations of factors that can alleviate that suffering. Self-compassion and emotion regulation are multidimensional constructs that can reduce the impact of negative views of the self and enhance effective coping in PTSD (Braehler & Neff, 2020; Kindred et al., 2024; Winders et al., 2022). Both constructs are also related to each other and to alexithymia (Kindred et al., 2024).

### 3.1. Self-Compassion, Alexithymia and Symptoms of PTSD

Self-compassion is generally defined as the ability to recognise one’s own suffering combined with a strong commitment to relieve that suffering (Gilbert, 2014). Neff (2003) drew upon aspects of Buddhist philosophy to identify three dimensions of compassionate self-related responses to stressful experiences. These include: (a) invoking self-kindness and understanding rather than critical self-judgement; (b) recognising suffering as part of the general human experience rather than feeling isolated; and (c) adopting a balanced approach rather than overidentifying with suffering. Neff characterised self-compassion as maintaining a delicate balance between increased compassionate and reduced uncompassionate self-responding at times of personal suffering (Neff et al., 2018). Deficits in self-compassion have been linked to alexithymia and symptoms of PTSD.

An inverse relationship is evident between alexithymia and self-compassion. Thus, the emotional difficulties inherent in alexithymia that restrict emotional awareness obstruct the ability to engage in self-compassionate behaviours (Lyvers et al., 2020). Moreover, a systematic review by Miethe et al. (2023) established that, without emotional insight, people with alexithymia may experience heightened self-criticism and rumination, both known PTSD risk factors. Conversely, however, research on therapies that promote self-compassion shows that such interventions expand self-understanding and emotional insight and thereby reduce alexithymia (Neff, 2023).

Research also shows that self-compassion can buffer against PTSD symptoms (Braehler & Neff, 2020; Zeller et al., 2015). A systematic review by Winders et al. (2022) found 13 papers that reported correlations between self-compassion and PTSD symptoms using DSM diagnostic criteria. Of those studies, 11 reported moderate-to-strong negative correlations and two found moderate negative correlations. Thus, increased self-compassion is strongly associated with lower PTSD symptoms.

Eight studies identified by Winders et al. (2022) also examined correlations with the symptom subtypes. Here, relationships were more varied. Avoidance symptoms were significantly correlated with self-compassion in all studies, although the level of correlation varied from −0.16 to −0.65. Re-experiencing and alterations in arousal and reactivity were only significant in five studies (re-experiencing; *r* = −0.14 to −0.43; alterations in arousal and reactivity, *r* = −0.21 to −0.63) using *DSM-IV* criteria. Winders et al. (2022) did not include any studies that specifically investigated the negative alterations in the cognitions and mood symptom cluster of the *DSM-5*. However, Brier et al. (2023) found that self-compassion was more strongly associated with negative alterations in cognitions and mood than with re-experiencing and avoidance. In sum, the research shows that while total self-compassion is consistently related to PTSD symptoms, its relationship with the symptom subtypes is less well understood.

### 3.2. Emotional Regulation, Alexithymia, Self-Compassion and Symptoms of PTSD

Self-compassion reflects attitudes toward the self in times of stress which motivate the individual to adopt coping strategies. In contrast, emotion regulation focusses on the neural cognitive and behavioural processes involved in managing emotional experiences (Kindred et al., 2024). The processing of emotions involves selection of specific strategies to influence emotions (e.g., cognitive appraisal, expressive suppression) and is influenced by the person’s capacity to regulate emotions effectively (Seligowski et al., 2015; Tull et al., 2020). Research on emotion regulation in alexithymia and PTSD shows that both alexithymia and PTSD are associated with deficits in emotion regulation.

Alexithymia is connected to disturbed emotional regulation because people with alexithymia employ more strategies of disengagement with emotional stimuli than strategies of engagement (Mehta et al., 2025). Whereas engagement strategies involve further processing of emotional responses (e.g., cognitive reappraisal), disengagement strategies inhibit or divert the mental processing of emotions (Scheppes et al., 2014). In alexithymia, such disengagement leads to the more frequent use of strategies of withdrawal, ignoring or denying emotional reactions, behavioural disengagement, or substance abuse (Preece et al., 2023). Thus, people higher on alexithymia avoid focussing on their negative emotions. This means that they do not integrate emotional information into mental models of emotion, which would facilitate emotion regulation (Preece et al., 2018).

There is also evidence for a relationship between difficulties in emotional regulation and PTSD symptoms. Effective emotional regulation allows individuals to process traumatic experiences and manage trauma-related emotions without becoming overwhelmed. Difficulties in regulating negative emotions, such as fear, anger and sadness, intensify PTSD symptoms, including alterations in arousal and reactivity, avoidance and re-experiencing (Kindred et al., 2024). Gratz and Roemer (2004) developed a measure of difficulties in regulating negative emotions (DERS-N). Within this measure they specified six subscales measuring different types of difficulties. These included nonacceptance of negative emotional responses; difficulties maintaining goal-directed behaviour and impulsivity when distressed. Other factors comprised perceived limited access to emotion regulation strategies; and a lack of clarity about the emotion being experienced.

Difficulties regulating negative emotions have a somewhat reciprocal relationship with PTSD symptoms. Importantly, these difficulties constitute a vulnerability factor for PTSD. For example, Bardeen et al. (2013) found that pre-existing difficulties regulating negative emotions predicted the onset of PTSD. In a female university student sample, they collected DERS-N data prior to a shooting on campus. Higher DERS-N scores were found to predict higher PTSD symptoms immediately after the shooting and around eight months later. As such, difficulties in regulating negative emotions functioned as a predisposing and maintaining factor for PTSD symptoms after a traumatic event. Alternatively, the intense emotional distress associated with the symptoms of PTSD, such as re-experiencing the trauma and intense negative emotions, including fear and horror of shame, place massive demands on emotion regulation, and this can create such difficulties (Seligowski et al., 2015).

PTSD symptoms can be further exacerbated by deficits in the capacity to regulate positive emotions. Weiss et al. (2018) found that higher levels of PTSD symptoms were evident in people who could not accept feeling positive emotions. These people also showed difficulties in implementing goal-directed behaviours and in controlling impulsive behaviours when experiencing positive emotions. Weiss et al. (2020) suggested that problems with positive emotions may stem from a general fear of the autonomic arousal originally associated with trauma. Thus, heightened arousal linked to triggers of trauma symptoms can extend to other stimuli eliciting arousal, such as positive emotions,

Deficits in emotional regulation are also linked to self-compassion in determining how individuals process and respond to stress and trauma. Inwood and Ferrari (2018) conducted a systematic review of studies on self-compassion and mental health conditions, including trauma, which assessed emotion regulation as a mediator. That review established that higher levels of self-compassion are associated with more effective emotional regulation strategies. In turn, these strategies contribute to lower symptoms of PTSD and other mental disorders, including depression and stress. For example, Barlow et al. (2017) found a sequential mediation pattern for PTSD symptoms. In their study, experiences of early childhood abuse were associated with higher levels of trauma symptoms and were partially mediated by self-compassion and emotion regulation. Thus, lower self-compassion was associated with greater difficulties in regulating negative emotions and subsequently higher levels of symptoms of PTSD.

## 4. The Current Study

**Figure 1:**
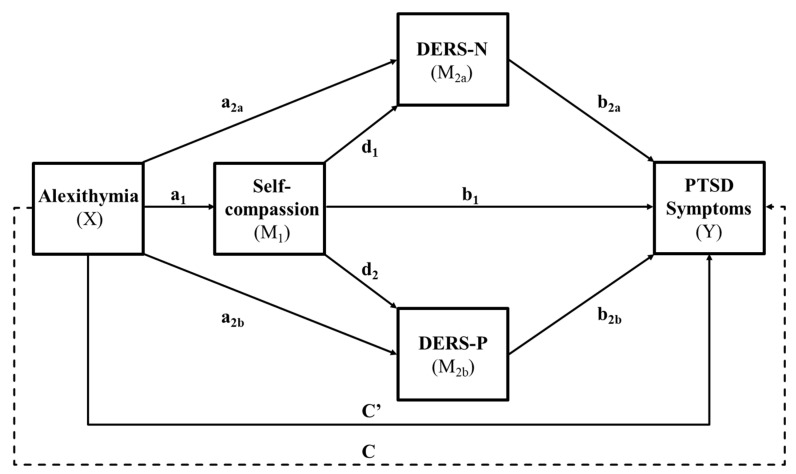
Conceptual process model. Note: ----- total effect total effect; –––– significant effect; DERS-N—Disturbed Emotional Regulation Scale—Negative; DERS-P—Disturbed Emotional Regulation Scale—Positive.

The primary aim of this study was to explore a model of the potential pathways between alexithymia and PTSD symptoms, focussing on the possible mediating roles of self-compassion and emotional regulation (both positive and negative). As depicted in Figure 1, alexithymia (X) was expected to be associated with lower self-compassion (M_1_), greater difficulties in regulating both negative and positive emotions (M_2a_ & M_2b_), and elevated symptoms of PTSD (Y). Consistent with Barlow et al. (2017), the mediators were expected to operate in a sequential pattern, with higher self-compassion relating to lower positive- and negative-emotion regulation difficulties and lower symptoms of PTSD. Both forms of difficulty in emotion regulation were expected to be associated with higher symptoms of PTSD. As an exploratory set of analyses, we also examined how the sequential model applied to the specific PTSD symptoms of re-experiencing, avoidance, negative alterations in cognitions or mood, and alterations in arousal and reactivity. Age and gender were included as covariates to establish that any relationships found were independent of their effects. This was because women are more likely to be diagnosed with PTSD than men, and trauma levels can differ across the lifespan (APA, 2022). A university sample was chosen given the higher-than-average levels of alexithymia among people aged 18–30 (Lyvers et al., 2020).

Three hypotheses and one research question were investigated. The hypotheses were:

**Hypothesis** **1:** *Alexithymia will be associated with higher levels of PTSD symptoms.*

**Hypothesis** **2:** *Alexithymia will be associated with lower self-compassion and greater difficulties regulating positive and negative emotions.*

**Hypothesis** **3:** *Lower self-compassion and higher disturbed emotional regulation (negative and positive) will sequentially mediate the relationship between alexithymia and PTSD symptoms. Specifically, higher levels of alexithymia will be related to lower levels of self-compassion, which, in turn, will be associated with more disturbed negative and positive emotional regulation, ultimately contributing to higher levels of PTSD symptoms.*

The research question was:

“Do self-compassion and emotional regulation difficulties (negative and positive) sequentially mediate the relationship between alexithymia and specific PTSD symptoms, including re-experiencing, avoidance, negative alterations in cognitions and mood and alterations in arousal and reactivity?”

## 5. Method

### 5.1. Participants and Procedure

This study used a convenience sample comprising students from Swinburne University of Technology and people from the community. Only individuals over 18 years of age capable of speaking and understanding English participated (*M* = 27.18; *SD* = 10.38; range: 18 to 65). The final sample included 332 participants after removal of 95 people who completed less than 30% of the online questionnaire and 9 univariate outliers and 1 multivariate outlier. Of these, 315 were Swinburne undergraduate students (82 male, 225 female, 8 non-binary) and 17 were community members (3 male, 11 female, 3 non-binary). Most participants were female (71.1%) and studying full time (65.66%) (see Table 1). Employment statuses were diverse, with participants spread across the full-time, part-time, casual and unemployed categories.

Although we used a non-clinical community sample, we found that many people in the sample currently reported high symptoms of PTSD. Of the total sample, 161 (48.9%) participants exceeded the clinical cut-off of 33 or greater on the PTSD Checklist for the *DSM-5* (PCL-5) (Weathers et al., 2013 [described in measures]). A third of the sample also reported having received a mental health diagnosis (33.5%) and/or psychiatric treatment (31.6%) at some point in their lifetime. This suggests that our sample included several people who had experienced substantial symptoms in response to potentially traumatic events. The events nominated by participants as part of the PCL-5 covered a wide variety of serious potentially traumatic events. These included sexual and physical assault, abusive relationships and domestic violence, life-threatening events and witnessing the death and injury of others.

Participants completed the research questionnaire online in Qualtrics and were asked to complete all tasks on one occasion, taking approximately 60 min. Participants were recruited from social media platforms (Instagram, Facebook) and Swinburne University’s Research Experience Program (REP). They received the questionnaire link through an advertisement on their respective platforms to voluntarily take part in this study. Swinburne students enrolled in the REP received course credit after completing the survey. No compensation was offered to community participants.

An information statement at the beginning of the survey detailed the purpose of this study and explained the implied consent process. It also provided information about support services available for anyone who might have responded negatively after completing the survey. As part of the survey, participants first completed the demographic items and then were prompted to identify and recall the most potentially traumatic event they had experienced. Following this, they responded to five standardised psychometric scales. Submitting the survey was taken as implied consent from the participants to include their data in this study. Participants could withdraw without penalty at any time before submission of their survey by closing the webpage. Participants were anonymous and non-identifiable. The project was approved by the Swinburne University Human Research Ethics Committee (2024-780918121). The data were collected between August 2024 and November 2024.

### 5.2. Measures

#### 5.2.1. Perth Alexithymia Questionnaire (PAQ) (Preece et al., 2018)

The PAQ is a 24-item self-report questionnaire measuring alexithymia, a personality construct characterised by difficulties in recognising and expressing negative and positive emotions. Its three subscales are: *Difficulty Identifying Feelings* (e.g., *when I am feeling bad/good I can’t make sense of those emotions*); *Difficulty Describing Feelings* (e.g., *When I am feeling bad/good I can’t talk about those feelings in much depth or detail*); and *Externally Oriented Thinking* (e.g., *I don’t try to be ‘in touch’ with my emotions*). Respondents rate each statement on a Likert scale ranging from 1 (*strongly disagree*) to 7 (*strongly agree*), where the total score of all items ranges from 24 to 168. Higher scores indicate greater difficulties in identifying and describing emotions, with the added strength of assessing both positive and negative emotional experiences. The psychometric properties of the PAQ are firmly established, with high levels of internal consistency (α = 0.75–0.98), test–retest reliability (*r* = 0.79–0.87) and discriminant validity (*r* = 0.60–0.82) demonstrated by various authors across different cultural adaptations (Cai et al., 2024; Lashkari et al., 2021; Preece et al., 2018).

#### 5.2.2. The PTSD Checklist for the *DSM-5* (PCL-5) (Weathers et al., 2013)

Before completing the PCL-5, participants reflected on and identified their most traumatic experience, and they completed the survey with that specific event in mind. The PCL-5 assessed the presence and severity of PTSD symptoms in the *DSM-5-TR* (APA, 2022), encompassing 20 items rated on a Likert scale from 0 (*not at all*) to 4 (*extremely*). Total scores range from 0 to 80, with higher scores indicating more severe PTSD symptoms. A threshold score of 33 or above is indicative of probable PTSD. The PCL-5 evaluates core PTSD symptom clusters, including *re-experiencing* (e.g., *repeated, disturbing, and unwanted memories of the stressful experience*); *avoidance* (*avoiding memories, thoughts, or feelings related to the stressful experience*, *negative changes in mood and cognition* (e.g., *having strong negative feelings, such as fear, horror, anger, guilt, or shame*)); and *hyperarousal* (e.g., *feeling jumpy or easily startled*). *It* has demonstrated strong psychometric properties, with high internal consistency (α ≈ 0.94), good test–retest reliability (*r* ≈ 0.82) and solid convergent (*r* = 0.74–0.85) and discriminant (*r* = 0.31–0.60) validity (Blevins et al., 2015; Hoeboer et al., 2024; Ibrahim et al., 2018).

#### 5.2.3. Self-Compassion Scale (SCS) (Neff, 2003)

The SCS is a 26-item self-report questionnaire that assesses individuals on six subscales of self-compassion reflecting their typical reactions to difficult experiences or feelings about themselves. Three of these subscales reflect compassionate self-responding: *Self-Kindness* (e.g., *I try to be understanding and patient towards those aspects of my personality I don’t like*); *Common Humanity* (e.g., *When I’m down, I remind myself that there are lots of people in the world feeling like I am*); *and Mindfulness* (e.g., *When something painful happens I try to take a balanced view of negatively the situation*). The other subscales include negatively worded items that are worded and reverse-scored to measure reduced uncompassionate self-responding. These scales include: *Self-Judgment* (e.g., *I’m disapproving and judgmental about my own flaws and inadequacies*); *Isolation* (e.g., *When I’m really struggling, I tend to feel like other people must be having an easier time of it*); and *Overidentification* (e.g., *When I’m feeling down I tend to obsess and fixate on everything that’s wrong*). Respondents rate each statement on a Likert scale from 1 (*almost never*) to 5 (*almost always*), and the total is the average of all 26 items. Higher scores indicate greater self-compassion. The psychometric properties of the self-compassion scale are robust, with high levels of internal consistency (α = 0.77–0.92), test–retest reliability (*r* = 0.88–0.93) and discriminant validity (*r* = −0.47–0.59) demonstrated by various authors (Büyüköksüz et al., 2025; Neff, 2003).

#### 5.2.4. The Difficulties in Emotion Regulation Scale—Negative (DERS-N) (Gratz & Roemer, 2004)

The DERS-N is a 36-item self-report questionnaire assessing difficulties in regulating negative emotions, with 11 of the items being reverse-scored. It comprises six subscales: *Nonacceptance of Emotional Responses* (e.g., *When I’m upset, I become irritated at myself for feeling that way*); *Difficulties Engaging in Goal-Directed Behaviour* (e.g., *When I’m upset, I have difficulty getting work done*); *Impulse Control Difficulties* (e.g., *When I am upset I lose control over my behaviours*); *Lack of Emotional Awareness* (e.g., *I pay attention to how I feel -reverse scored*); *Limited Access to Emotion Regulation Strategies* (e.g., *When I am upset*, I believe there is nothing I can do to make myself feel better); and *Lack of Emotional clarity* (e.g., *I am confused about how I feel*). Items are rated on a 5-point Likert scale, ranging from 1 (*almost never*) to 5 (*almost always*), and 11 items are reverse-scored. A total score of all subscales ranges from 36 to 180, with higher scores indicating greater difficulties in emotion regulation. The psychometric properties of the DERS-N are well established, with high levels of internal consistency (α = 0.84–0.95), test–retest reliability (*r* = 0.85–0.88) and discriminant validity demonstrated across multiple studies (Bjureberg et al., 2016; Hallion et al., 2018).

#### 5.2.5. The Difficulties in Emotion Regulation Scale—Positive (DERS-P) (Weiss et al., 2019)

The DERS-P is a 13-item self-report questionnaire adapted from the original DERS (Gratz & Roemer, 2004) to assess difficulties specifically related to regulating positive emotions. It includes three subscales: *Nonacceptance of Positive Emotions* (e.g., *When I’m happy, I feel guilty for feeling that way*); *Difficulties Engaging in Goal-Directed Behaviour* (e.g., *When I’m happy, I have difficulty getting work done*); *Difficulties Controlling Impulsive Behaviours When Experiencing Positive Emotions* (e.g., *When I’m happy, I have difficulty controlling my behaviours*). Items are rated on a 5-point Likert scale, ranging from 1 (*almost never*) to 5 (*almost always*), where the total score of all subscales ranges from 13 to 65. Higher scores indicate greater difficulties in regulating positive emotions. The psychometric properties of the DERS-P are robust, with high levels of internal consistency (α = 0.90–0.94), test–retest reliability (*r* = 0.84–0.89) and discriminant validity demonstrated across multiple studies (Hallion et al., 2018; Weiss et al., 2015, 2019).

### 5.3. Data Analysis

Prior to conducting analyses, we screened the data set for missing values, outliers, and normality of distributions. Of an initial pool of 459 respondents, cases with 30% or more missing data were excluded (*n* = 95). After removal of these cases, no other respondents had any missing data.

The data were screened for univariate and multivariate outliers. Univariate outliers were identified by examining standardised z-scores, with values exceeding ±2.68 and outside the interquartile range (0.25 to 0.75). Nine outliers were detected and excluded. Multivariate outliers were detected through Mahalanobis distance within the SPSS Version 31 regression analysis. One case exceeded the chi-square distribution critical value for the four independent variables at a significance level of 0.001 and was excluded. The final data set included 332 participants, which exceeded the number determined as a minimum by power analysis. An a priori power analysis showed that a total sample size of at least 162 participants was required to attain a power of 0.8 in the Percentile Bootstrap Test under the small-to-medium size effect (0.26) (Fritz & MacKinnon, 2007).

Following the outlier assessment, the data were examined for skewness and kurtosis. Significant deviations from normality were taken to be indicated by skewness and kurtosis values exceeding ±2 (Tabachnick & Fidell, 2019). Overall, the data displayed normality, indicating that the distribution of the data points approximated a normal distribution curve. Therefore, no data transformations were required. Pearson product–moment correlations evaluated the strength and direction of the linear relationships among all measures, and homoscedasticity checks also appeared satisfactory, as all the scatter plot points were randomly and evenly dispersed around the zero-horizontal line, showing no clear pattern.

Multicollinearity was checked by inspection of the correlations and calculation of the tolerance and Variance Inflation Factor (VIF) scores for each of the four independent variables. Pearson product–moment correlations were above 0.7 for the DERS-N total scale with self-compassion (*r* = 0.78) and the PAQ (*r* = 0.71), indicating possible multicollinearity. To deal with this possibility, the score for the subscale Lack of Emotional Awareness was subtracted from the DERS-N total score to create a shortened version (DERS-N-S). This was based on the observation that the lack-of-awareness items are similar in content to alexithymia (e.g., When I’m upset, I acknowledge my emotions [reversed]) and thus might contribute to item overlap and multicollinearity. As there was no conceptual link between self-compassion and the DERS-N, the subscale scores for self-compassion were correlated with the DERS-N total score. That comparison revealed a high correlation for the overidentification-with-emotions subscale (*r* = −0.76). As this subscale includes items reflecting inability to manage negative emotions (e.g., When something upsets me, I get carried away with my feelings [Reversed]), it was seen as a possible source of content overlap. This subscale was therefore removed from the total SCS score to create a shortened variable (SCS-S). Creation of the two modified variables for the DERS-N total and SCS total reduced the correlations with alexithymia and the DERS-N-S to below 0.70 (DERS-N-S with alexithymia, *r* = 0.66; DERS-N-S with SCS-S, *r* = 0.68). After these two modifications, tolerance and VPI scores were calculated for the independent variables. All tolerance levels were above the required level of 0.1 (0.343 to 0.696), and all VIF coefficients were below the most conservative level of 3.0 (1.437 to 2.916). This suggested that there was no multicollinearity in the data. As shortened measures, the DERS-N_S and the SCS-S are not entirely comparable to the full-scale versions used in other studies. However, the correlations between the DERS-N-S and DERS-N and the SCS and SCS-S were both *r* = 0.984, suggesting that they are essentially duplicate measures.

As all our measures were self-report scales, we also tested for common method bias using Harman’s single-factor test. The percentage of variance accounted for by all the test items on a single factor was 30.84%. We concluded that there was no indication of common method variance because this value is well below the 50% variance level set to determine possible bias.

A serial mediation analysis using PROCESS Version 4.2 (Model 81) from Hayes (2022) in SPSS was conducted to test direct and indirect effects of a predictor (X—alexithymia) on an outcome variable (Y—PTSD symptoms) through multiple mediators: self-compassion (M_1_) and then emotional regulation (disturbed negative-emotion regulation (M_2a_) and disturbed positive-emotion regulation (M_2b_)) (see Figure 1, p. 7). The analysis employed 5000 bootstrapped samples and percentile-based 95% confidence intervals (CIs) to assess the indirect effects of the predictor variables on the outcome variable via the mediator variables. Direct effects were deemed significant when the alpha level was below 0.05, while indirect effects were considered significant if the confidence intervals did not cross zero (Hayes, 2022).

## 6. Results

The descriptive statistics and intercorrelations among the variables are provided in Table 2. Table 2 shows that there were significant correlations among all the main variables, and that all measures showed excellent internal consistency (the Cronbach’s alpha ranged from 0.84 to 0.96). Higher alexithymia traits were strongly correlated with higher levels of PTSD symptoms, and with lower levels of self-compassion. Participants with high levels of alexithymia traits also experienced more difficulties in regulating negative and positive emotions. Conversely, self-compassion showed negative correlations with all the other variables. In terms of the covariates, age was modestly positively correlated with self-compassion and had modest negative correlations with alexithymia and difficulties in emotion regulation. It did not correlate with the PTSD symptom measures. For gender, being female was modestly correlated with the PTSD symptom measures but was uncorrelated with alexithymia, self-compassion and emotion regulation difficulties.

### 6.1. Serial Mediation Model for Total PTSD Symptoms

**Figure 2:**
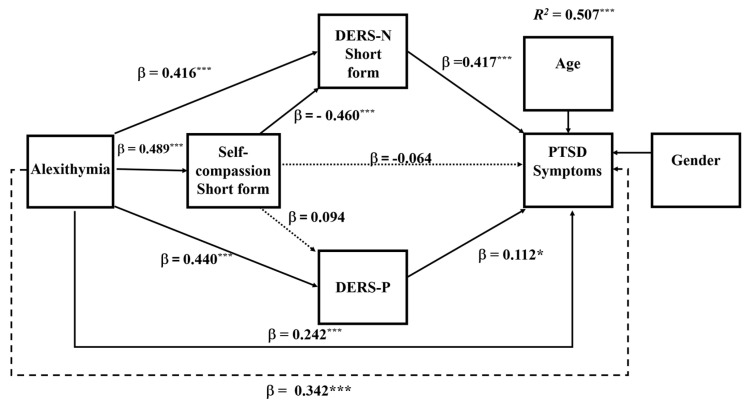
Serial Mediation Model: Total PTSD Symptom Results. Notes: * *p* < 0.05; *** *p* < 0.001; ----- total effect; ⋯⋯ nonsignificant effect; –––– significant effect; DERS-N Short form—Difficulties in Emotional Regulation Scale—short form; DERS-P—Difficulties in Emotional Regulation Scale—Positive. β—standardised coefficient.

A serial mediation model was performed using PROCESS Version 4.2 (Model 81) from Hayes (2022) in SPSS, with alexithymia as the predictor, self-compassion, emotional regulation (positive and negative) as the mediators and Post-Traumatic Stress Disorder symptoms as the dependent variable. Results are illustrated in Figure 2.

The overall model was significant (*R*^2^ = 0.507, *F*(6, 314) = 53.79, *p* < 0.0001), explaining = 51% of the variance in PTSD symptoms. As shown in Figure 2, as hypothesised (Hypothesis 1), alexithymia directly predicted PTSD symptoms (β = 0.242, *SE* = 0.033, *p* < 0.0001), and its relationship with PTSD symptoms was partially mediated. There was also support for the second hypothesis, as alexithymia significantly predicted lower self-compassion (β = −0.489, *SE* = 0.001, *p* < 0.0001) and greater difficulties in regulating both negative (β = 0.416, *SE* = 0.032, *p* < 0.0001) and positive (β = 0.440, *SE* = 0.016, *p* < 0.0001) emotions, demonstrating its strong influence on emotional regulation and self-compassion.

There was partial support for the third hypothesis, with the sequential mediation pathway through self-compassion and difficulties regulating negative emotions being significant (*B* = 0.094, *BootSE* = 0.019, *BootCI* [0.059, 0.134]). This indicates that higher alexithymia is associated with lower self-compassion, greater difficulties regulating negative emotions and increased total PTSD symptoms. However, the sequential pathway through difficulties in regulating positive emotions was nonsignificant (*B* = −0.003, *BootSE* = 0.002, *BootCI* [−0.009, 0.001]). Additionally, self-compassion was not a significant single mediator of alexithymia and total PTSD symptoms (*B* = 0.019, *BootSE* = 0.018, *BootCI* [−0.015, 0.056]) and was not associated with difficulties in regulating positive emotions (*B* = 0.231, *BootSE* = 0.144, *BootCI* [−0.051, 0.514]). However, difficulties in regulating negative emotions and difficulties in regulating positive emotions were significant single mediators of alexithymia (DERS-N-S: *B* = 0.173, *BootSE* = 0.035, *BootCI* [0.110, 0.243]; DERS-P: *B* = 0.049, *BootSE* = 0.022, *BootCI* [0.009, 0.096]). All coefficients and confidence levels for all effects are presented in Table 3.

### 6.2. Mediation Analysis of Specific PTSD Symptom Subtypes

**Figure 3:**
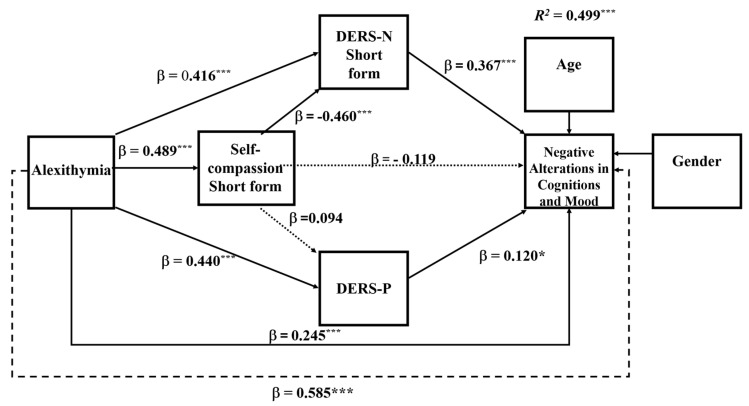
Serial Mediation Model 81: PTSD Symptoms for Negative Alterations in Cognitions and Mood Symptoms of PTSD. Notes: * *p* < 0.05; *** *p* < 0.001; ----- total effect; ⋯⋯ nonsignificant effect; –––– significant effect; DERS-N—difficulties in regulating negative emotions—short form; DERS-P—difficulties in regulating positive emotions. β—standardised coefficient.

**Figure 4:**
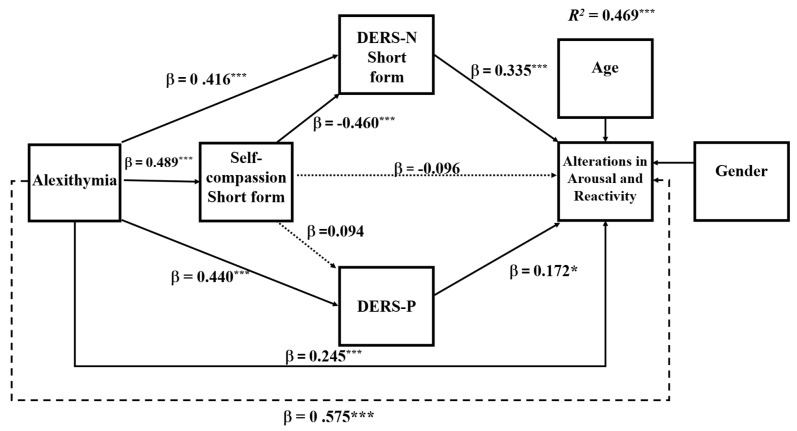
Serial Mediation Model 81: PTSD Symptoms for Alterations in Arousal or Reactivity Symptoms of PTSD. Notes: * *p* < 0.05; *** *p* < 0.001; ----- total effect; ⋯⋯ nonsignificant effect; –––– significant effect; DERS-N—difficulties in regulating negative emotions; DERS-P—difficulties in regulating positive emotions. β—standardised coefficient.

**Figure 5:**
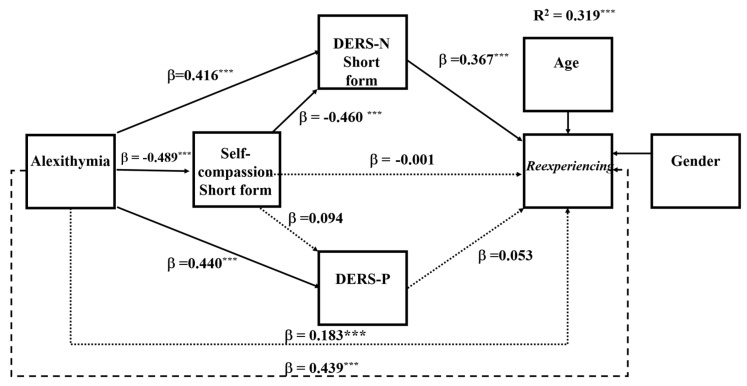
Serial Mediation Model 81: PTSD Re-experiencing Symptoms. Note: *** *p* < 0.001; ----- total effect; ⋯⋯ nonsignificant effect; –––– significant effect; DERS-N-S—difficulties in regulating negative emotions—short form; DERS-P—difficulties in regulating positive emotions. β—standardised coefficient.

**Figure 6:**
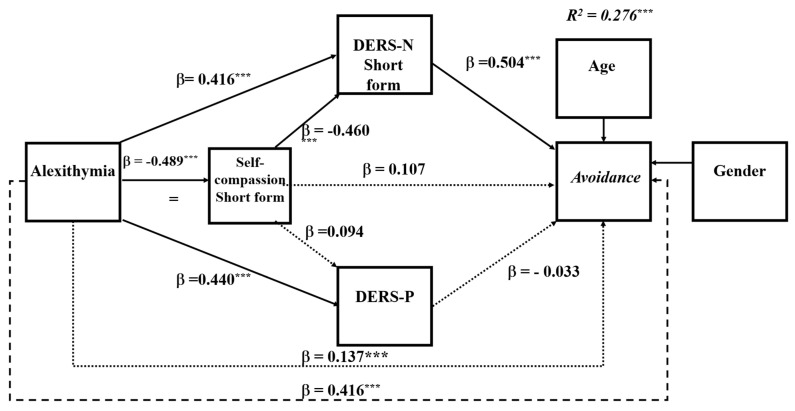
Serial Mediation Model 81: PTSD Avoidance Symptoms. Note: *** *p* < 0.001; ----- total effect, ⋯⋯ nonsignificant effect; –––– significant effect; DERS-N–S—difficulties in regulating negative emotions—short form; DERS-P—difficulties in regulating positive emotions. β—standardised coefficient.

To explore the research question, the same mediation analysis was repeated for each of the PTSD symptom clusters. Analyses of the specific symptoms showed that in all symptom clusters, the DERS-N-S was a consistent mediator of alexithymia and self-compassion and self-compassion’s influence on symptoms operated mainly through the DERS-N-S. The DERS-P was a significant mediator primarily for negative alterations in cognitions and mood and alterations in arousal and reactivity. Overall, negative alterations in cognitions and mood and alterations in arousal and reactivity showed the same pattern of results as for that for the total PTSD symptoms (see Figure 3 and Figure 4). However, for symptoms of re-experiencing and avoidance, difficulties in regulating positive emotions was no longer a significant single mediator (see Figure 5 and Figure 6). The coefficients and confidence levels for all effects related to each symptom type are tabled in Supplementary File S1.

### 6.3. Serial Mediation Analyses for Negative Alterations in Cognitions and Mood and Alterations in Arousal and Reactivity

The overall model for negative alterations in cognitions and mood was significant (*R*^2^ = 0.499, *F*(6, 314) = 29.423, *p* < 0.0001), explaining = 49.88% of the variance. The overall model for alterations in arousal and reactivity was also significant (*R*^2^ = 0.469, *F*(6, 314) = 46.122, *p* < 0.0001), explaining = 46.85% of the variance. Alexithymia directly predicted negative alterations in cognitions and mood and alterations in arousal and reactivity and so was partially mediated in both sets of analyses (negative alterations in cognitions and mood: *B* = 0.059, *SE* = 0.013, *BootCI* [0.000, 0.033]; alterations in arousal and reactivity: *B* = 0.048, *SE* = 0.011, *BootCI* [0.026, 0.070]).

### 6.4. Serial Mediation Analyses for Re-Experiencing and Avoidance Symptoms of PTSD

The overall model for re-experiencing was significant (*R*^2^ = 0.32, *F*(6, 314) = 24.509, *p* < 0.0001), explaining = 31.90% of the variance in re-experiencing. Similarly, the overall model for avoidance was significant (*R*^2^ = 0.276, *F*(6, 314) = 19.91, *p* < 0.0001), explaining 27.56% of the variance. Alexithymia directly predicted re-experiencing and avoidance symptoms and so was partially mediated in both sets of analyses (re-experiencing, *B* = 0.31, *SE* = 0.011, *BootCI* [0.004, 0.010]; avoidance symptoms, *B* = 0.011, *SE* = 0.005, *BootCI* [0.038, 0.006]). As shown in Figure 4 and Figure 5, the only difference in these models was the absence of the mediation of alexithymia by difficulties in regulating positive emotions.

## 7. Discussion

This study was an initial investigation of a model of the relationship between alexithymia and symptoms of PTSD and the potential mediating roles of self-compassion and emotion regulation (both positive and negative). As an exploratory investigation, this model was also evaluated in relation to the four subtypes of PTSD symptoms (re-experiencing, avoidance, negative alterations in cognitions and mood, and alterations in arousal and reactivity). Controlling for age and gender, our findings were broadly as expected and generally support the model we developed. Alexithymia was confirmed as significantly related to higher PTSD symptoms, both directly and through various indirect pathways. As hypothesised (Hypothesis 1), higher alexithymia was directly associated with higher levels of total PTSD symptoms. Also as hypothesised (Hypothesis 2), higher alexithymia was also associated with lower self-compassion and greater difficulties in regulating negative and positive emotions. In partial support of Hypothesis 3, sequential mediation was evident for self-compassion and difficulties regulating negative emotions. Higher alexithymia was associated with lower self-compassion, and lower self-compassion was associated with greater difficulties regulating negative emotions, which was related to higher symptoms of PTSD. Contrary to expectation, sequential mediation was not evident for difficulties with positive-emotion regulation, as the pathway from self-compassion to difficulties with positive emotions was nonsignificant. However, alexithymia was significantly associated with difficulties regulating positive emotions, and difficulties regulating positive emotions, in turn, were associated with higher levels of PTSD symptoms. Further, self-compassion was not directly associated with the total PTSD symptoms. Its only association with the PTSD symptoms was through its association with difficulties in regulating negative emotions. The results thus highlight self-compassion and negative- and positive-emotion regulation as partial sequential mediators of alexithymia’s association with higher PTSD symptoms.

Exploration of the relationship between alexithymia and the different PTSD symptom subtypes revealed variability in how emotional regulation difficulties and self-compassion mediated the effects of alexithymia. The models for symptoms of negative alterations in cognitions and mood and alterations in arousal and reactivity duplicated the pattern of prediction for the total PTSD symptoms. However, differences emerged for the symptoms of re-experiencing and avoidance. In these models, difficulties in regulating positive emotions were not associated with either symptom type. In this discussion, we consider the theoretical and clinical implications of the findings for the total PTSD symptoms and for the four symptom subtypes.

### 7.1. Alexithymia and PTSD Symptoms

Our findings clearly show that alexithymia is directly associated overall and with all the subtypes of PTSD symptoms. The analyses also show that alexithymia has indirect associations with PTSD symptoms via self-compassion and difficulties regulating both negative and positive emotions. This is consistent with past research that has established alexithymia as a vulnerability and maintenance factor in PTSD (e.g., Edwards, 2022; Luminet & Nielson, 2025). Clearly, difficulties in identifying and expressing emotions and a focus on external stimuli rather than internal emotions are associated with the impaired processing of psychologically traumatic events. Therapeutically, this reinforces the need for clinicians treating PTSD to determine the client’s level of alexithymia.

Our analysis of the symptom subtypes confirmed the strong indirect pathways for self-compassion and difficulties in regulating negative emotions for PTSD symptoms overall. This aligns with the general pattern of findings for self-compassion and difficulties in regulating negative emotions reported in Inwood and Ferrari’s (2018) systematic review. Thus, self-compassion appears to act more as a motivational approach to problem solving (Bates et al., 2021; Finlay-Jones et al., 2015) and is associated with fewer specific problems with negative-emotion regulation (e.g., impulsivity, perceived limited access to strategies). This is also consistent with Preece et al. (2023), who found that people with alexithymia overutilise less effective disengagement strategies (e.g., denial, distraction) and underutilise effective engagement strategies (e.g., problem solving). Taken together, this suggests that difficulties regulating negative emotions is an important mechanism in determining the effects of alexithymia and self-compassion on symptoms of PTSD.

Difficulties in regulating positive emotions also mediated the relationship between alexithymia and the overall PTSD symptoms. This relationship was also evident for the symptom subtypes of negative alterations in cognitions and mood and alterations in arousal and reactivity. The association with negative alterations in cognitions and mood may stem from the inclusion of symptoms of emotional numbing within this symptom subtype. In particular, the *DSM-5-TR* symptom referencing a persistent inability to experience positive emotions (e.g., happiness, satisfaction) is likely to be linked to difficulties regulating positive emotions, such as nonacceptance of positive emotions. That difficulties regulating positive emotions are also related to symptoms of alterations in arousal and reactivity aligns with a suggestion by Weiss et al. (2020). They argued that problems in accepting positive emotions, and in implementing goal-directed behaviours and avoiding impulsive actions, reflect a general fear of elevated arousal. In PTSD, this fear is heightened when arousal is triggered by traumatic events and is connected to elevated resting arousal experienced through feeling superalert, jumpy or easily startled states (DSM-5-TR, 2022).

Our analyses also showed that difficulties in regulating positive emotions did not mediate symptoms of re-experiencing or avoidance. Re-experiencing involves intense, focused affect linked to a traumatic event, and avoidance involves behavioural, cognitive and emotional attempts to escape distress. These consciously mediated experiences focus on the triggered reminiscence of the traumatic event and may be more closely linked to regulating negative emotions. This includes nonacceptance of the negative emotion and difficulties in maintaining goal-directed behaviour when distressed (Gratz & Roemer, 2004; Kindred et al., 2024). Similarly, the elements of self-compassion, such as overidentification with suffering or keeping a balanced mind (mindfulness), may be important in determining the influence of alexithymia on these symptoms. These possibilities clearly warrant attention in future work.

### 7.2. Self-Compassion and PTSD Symptoms

Contrary to expectations, self-compassion did not relate directly to the total symptoms of PTSD or any of the PTSD symptom subtypes. Instead, its influence on the total symptoms of PTSD was indirect via its association with difficulties regulating negative emotions. This is surprising given that earlier research shows a relationship between self-compassion and PTSD symptoms (Braehler & Neff, 2020; Winders et al., 2022). The absence of a direct relationship may reflect differences in the contributions to the association with PTSD symptoms among the components of self-compassion. MacBeth and Gumley (2012) demonstrated that the components of reduced uncompassionate behaviour (i.e., self-judgment, isolation and overidentifying with feelings) were more strongly related to symptoms of depression and anxiety than were the compassionate components (i.e., self-kindness, common humanity and mindfulness). This also seems to be the case for other types of symptoms, such as social anxiety (e.g., Bates et al., 2021; McBride et al., 2022). Combining the two forms of symptoms as a total score may, therefore, mask direct effects for some facets of self-compassion with PTSD symptoms. To explore this possibility, future research utilising the SCS could examine self-compassion as a bifactor model divided into compassionate self-responding and reduced uncompassionate self-responding. This would allow for an examination of differences in the influence of positive compassionate actions in contrast to the reduction in negative uncompassionate responses to the self. Alternatively, differences among the six individual subscales of self-compassion could be investigated to further localise any direct relationships.

Although self-compassion was strongly associated with lower difficulties in regulating negative emotions, it was not associated with difficulties regulating positive emotions. This difference may reflect the nature of self-compassion. Self-compassion is defined as a general capacity to detect suffering in oneself and a desire to alleviate suffering (Gilbert, 2014). This focus on suffering is, therefore, much more likely to be activated by negative emotion than positive emotion. Although the DERS-P does incorporate negative feelings into responses to positive emotions (e.g., guilt), the context is in times of feeling happy. Qualitative analysis of in-depth interviews of specific instances of difficulties in regulating positive emotions may be needed to elucidate the association with self-compassion.

### 7.3. Limitations and Directions for Future Research

The present study is not without limitations. First, the use of a convenience sample, predominantly comprising female undergraduate students, limits the generalisability of the results. While we controlled for the influence of age and gender, our sample may not represent broader populations. Further, although almost half of our sample scored above the cut-off for PTSD on the PCL-5, we did not have ethics clearance to gather full information on the trauma experiences of the participants. We, therefore, did not control the types of traumas, numbers of traumatic events or the time since the traumatic events to which the participants referred. Future research is needed to take full account of participants’ levels of trauma exposure and differences in demographic backgrounds. Research on clinical samples and occupational groups exposed to traumatic experiences (e.g., health care workers, defence force personnel, or police) would also help determine the generalisability of our data.

A second limitation is our cross-sectional design. The present mediation analysis identified theoretically meaningful patterns of association and mediation amongst the variables. Longitudinal data is required, however, to confirm the causal relationships among alexithymia, emotional regulation difficulties, self-compassion and PTSD symptoms. Treatment outcome studies will also be important in establishing how these variables change over time and contribute to trauma recovery. As some research suggests that increased alexithymia can also be an outcome of PTSD (Kindred et al., 2024), cross-lagged analyses would help determine the nature of the reciprocal relationship between alexithymia and PTSD symptoms. Another extension of this study would be to include measures of general distress in the analysis, which would allow for the differentiation of the relationships with symptoms of PTSD from general indicators of emotional distress (e.g., depression or anxiety).

A final limitation of this study relates to our reliance on self-report measures. The measures we used are well-validated and relatively free of social desirability concerns. Nevertheless, they are subject to the limitations of self-report measures. While the Harman single-factor test did not suggest common method variance, this broad test cannot preclude common method variance (Podsakoff et al., 2003). Latent variable analysis (e.g., structural equation modelling) with marker variables would offer more specific control of common method variance. Multi-method analysis including interview measures of PTSD symptoms and physiological assessments would also increase confidence in the findings. Qualitative analysis of interview data would further elucidate the lived experience of difficulties in regulating negative and positive emotions and self-compassionate self-responding in the context of alexithymia and PTSD. Finally, although our shortened measures of the DERS-N and the SCS were essentially equivalent to the full measures (*r* = 0.984), these remain customised for our study. They may not be comparable to full measures. Other ways of controlling multicollinearity without removing items (e.g., latent variable analysis) could be employed to replicate our findings.

Research is also needed to extend our finding that difficulties in regulating negative and positive emotions mediated the relationship between alexithymia and PTSD symptoms. A feature of the attention-and-appraisal model is its division of alexithymia into deficits specific to positive as well as negative emotions (Preece et al., 2018). Exploring these specific pathways would help clarify how such deficits influence difficulties in regulating emotions and increase our understanding of the attention and appraisal components of alexithymia specific to different emotions (Luminet & Nielson, 2025). This research could explore how alexithymia and difficulties in emotion regulation are influenced by PTSD symptoms and maintain each other in feedback loops.

### 7.4. Conclusions

In conclusion, the findings of this study contribute to the growing body of literature that positions alexithymia as associated with PTSD symptoms, primarily through its links with emotional regulation. We also found that self-compassion is part of a serial mediation pathway influencing PTSD symptoms through emotional regulation difficulties. Additionally, the data highlight the role of positive emotional regulation. This suggests that both positive and negative emotional regulation need to be targeted in trauma-focused therapies. The importance of addressing the difficulties in emotional regulation associated with alexithymia in clinical practice is further emphasised. Targeting both emotional regulation and self-compassion could improve therapeutic outcomes for individuals struggling with trauma. By integrating interventions that enhance emotional awareness and self-compassion (e.g., mindfulness, compassion-focussed therapy), clinicians may better address the broad spectrum of trauma-related symptoms, ultimately improving recovery outcomes for alexithymia patients. In the early phases of treatment, however, clients high in alexithymia with trauma-related fears of emotion or compassion may find direct work on self-compassion and emotional experience challenging. A first focus may need to be on establishing safety, building basic emotional awareness, and gradually introducing compassion-related exercises.

## Figures and Tables

**Table 1:** Summary of demographic information (*N* = 332).

	Participants
**Gender**	
Female	236
Male	85
Non-binary	11
**Employment**	
Full time	84
Part time	87
Casual	82
Not employed	79
**Study Status**	
Full time	218
Part time	106
Not studying	8
**Relationship Status**	
Single (never in a relationship)	43
Currently single	104
Married/de facto	65
In a relationship	110
Divorced	10
**Children**	
Yes	73
No	259
**Psychiatric Diagnosis (lifetime)**	
Yes	114
No	218
**Psychiatric Treatment (lifetime)**	
Yes	120
No	212

**Table 2:** Descriptive statistics and intercorrelations among variables.

Variable	*M*	*SD*	*Skew*	*Kurt*	Cronbach α	1	2	3	4	5	6	7	8	9	10	11
1. Alexithymia	84.91	31.61	0.06	−0.60	0.96	-										
2. Self-Compas.	14.18	3.6	−0.06	−0.54	0.92	−0.51 **	-									
3. DERS-N-S	82.50	24.81	0.06	−0.67	0.96	0.66 **	−0.68 **	-								
4. DERS-P	20.64	8.80	1.39	0.97	0.93	0.42 **	−0.15 **	0.47 **	-							
5. PTSD Total	32.83	19.37	0.20	−0.82	0.95	0.58 **	−0.48 **	0.67 **	0.40 **	-						
6. Re-experience	8.09	5.24	0.36	−0.67	0.88	0.44 **	−0.35 **	0.52 **	0.30 **	0.87 **	-					
7. Avoidance	4.11	2.54	−0.02	−1.20	0.84	0.39 **	−0.30 **	0.50 **	0.24 **	0.79 **	0.69 **	-				
8. NMC	11.76	7.65	0.19	−1.10	0.89	0.57 **	−0.51 **	0.66 **	0.38 **	0.93 **	0.72 **	0.68 **	-			
9. AAR	8.85	6.20	0.38	−0.71	0.87	0.57 **	−0.46 **	0.63 **	0.42 **	0.91 **	0.71 **	0.64 **	0.80 **	-		
10. Age	27.18	10.38	1.40	1.36	-	−0.19 **	0.15 *	−0.22 **	−0.20 **	−0.06	−0.06	−0.05	−0.07	−0.04	-	
11. Gender	-	-	-	-	-	−0.04	−0.05	0.10	−0.03	0.18 **	0.21 **	0.13 *	0.16 **	0.12 *	0.09	-

Note: * *p* < 0.05, ** *p* < 0.01, *N* = 332. Self-compas.: self-compassion short form; DERS-N-S: Difficulties in Emotion Regulation—Negative Emotions—short form; DERS-P: Difficulties in Emotion Regulation—Positive Emotions. NMC: negative alterations in cognitions and mood; AAR: alterations in arousal and reactivity.

**Table 3:** Total, indirect and direct effects of serial mediation with alexithymia and total PTSD symptoms with self-compassion (SCS-S), difficulties regulating negative emotions (DERS-N-S) and difficulties regulating positive emotions (DERS-P) as mediators.

	*B*	*SE*	*p*	LL	UL	ES
Total effect (of alexithymia on PTSD)	0.358	0.08	<0.0001	0.000	0.42	0.342
Indirect effect through SCS-S	0.019	0.02		−0.015	0.056	0.031
Indirect effect through DERS-N-S	0.106	0.02		0.067	0.151	0.173
Indirect effect through DERS-P	0.030	0.01		0.006	0.059	0.049
Indirect effect through SCS-S and DERS-N-S	0.057	0.01		0.036	0.083	0.937
Indirect effect through SCS and DERS-P	−0.003	0.00		−0.001	0.001	−0.001
Direct effect (*c′*)	0.148	0.03	<0.0001	0.083	0.214	0.242

Note. *B*, unstandardised coefficient. *SE*, bootstrap standard error. LL, bootstrap confidence interval lower limit. UL, bootstrap confidence interval upper limit. ES, standardised effect size.

## Data Availability

The raw data supporting the conclusions of this article will be made available by the authors upon reasonable request.

## Supplementary Materials

The following supporting information can be downloaded at: https://www.mdpi.com/article/10.3390/ejihpe16020030/s1, Table S1: Total, Indirect and Direct Effects of Serial Mediation: Alexithymia and Total PTSD Symptoms With Self-Compassion (SCS), Difficulties regulating Negative emotions (DERS-N and Difficulties Regulating Positive Emotions (DERS-P) as Mediators; Table S2: Total, Indirect and Direct Effects of Serial Mediation: Alexithymia and Re-experiencing PTSD Symptoms (ReX) With Self-Compassion (SCS), Difficulties regulating Negative emotions (DERS-N and Difficulties Regulating Positive Emotions (DERS-P) as Mediators; Table S3: Total, Indirect and Direct Effects of Serial Mediation: Alexithymia and Avoidance PTSD Symptoms With Self-Compassion (SCS), Difficulties regulating Negative emotions (DERS-N and Difficulties Regulating Positive Emotions (DERS-P) as Mediators; Table S4: Total, Indirect and Direct Effects of Serial Mediation: Alexithymia and Negative Alteration in mood and Cognition (NAMC) Symptoms With Self-Compassion (SCS), Difficulties Regulating Negative emotions (DERS-N and Difficulties Regulating Positive Emotions (DERS-P) as Mediators; Table S5: Total, Indirect and Direct Effects of Serial Mediation: Alexithymia and Altered Arousal and Reactivity (AAR) Symptoms With Self-Compassion (SCS), Difficulties regulating Negative Emotions (DERS-N and Difficulties Regulating Positive Emotions (DERS-P) as Mediators.

## References

[B1-ejihpe-16-00030] American Psychiatric Association [APA] (2022). Diagnostic and statistical manual of mental disorders *(5th ed., Text Revision, DSM-5-TR)*.

[B2-ejihpe-16-00030] Bardeen J. R., Fergus T. A., Orcutt H. K. (2013). Experiential avoidance as a moderator of the relationship between anxiety sensitivity and perceived stress. Behavior Research and Therapy.

[B3-ejihpe-16-00030] Barlow M. R., Goldsmith-Turow R. E. G., Gerhart J. (2017). Trauma appraisals, emotion regulation difficulties, and self-compassion predict posttraumatic stress symptoms following childhood abuse. Child Abuse & Neglect.

[B4-ejihpe-16-00030] Bates G. W., Elphinstone B., Whitehead R. (2021). Self-compassion and emotional regulation as predictors of social anxiety. Psychology and Psychotherapy.

[B5-ejihpe-16-00030] Bistricky S. L., Gallagher M. W., Roberts C. M., Ferris L., Gonzalez A. J., Wetterneck C. T. (2017). Frequency of interpersonal trauma types, avoidant attachment, self-compassion, and interpersonal competence: A model of persisting posttraumatic symptoms. Journal of Aggression, Maltreatment & Trauma.

[B6-ejihpe-16-00030] Bjureberg J., Ljótsson B., Tull M. T., Hedman E., Sahlin H., Lundh L. G., Bjärehed J., DiLillo D., Messman-Moore T., Gumpert C. H., Gratz K. L. (2016). Development and validation of a brief version of the difficulties in emotion regulation scale: The DERS-16. Journal of Psychopathology and Behavioral Assessment.

[B7-ejihpe-16-00030] Blevins C. A., Weathers F. W., Davis M. T., Witte T. K., Domino J. L. (2015). The posttraumatic stress disorder checklist for DSM-5 (PCL-5): Development and initial psychometric evaluation. Journal of Traumatic Stress.

[B8-ejihpe-16-00030] Braehler C., Neff K., Tull M. T., Kimbrel N. A. (2020). Self-compassion in PTSD. Emotion in posttraumatic stress disorder.

[B9-ejihpe-16-00030] Brier Z. M. F., Burt K. B., Legrand A. C., Price M. (2023). An examination of the heterogeneity of the relationships between posttraumatic stress disorder, self-compassion and gratitude. Clinical Psychology & Psychotherapy.

[B10-ejihpe-16-00030] Büyüköksüz E., Tekin I., Arıkan S., İlkay Ş., Erözkan A. (2025). Psychometric validation and measurement invariance of the self-compassion scale-short form (SCS-SF) across gender, clinical population, and cultures. BMC Psychology.

[B11-ejihpe-16-00030] Cai X. L., Ye Q., Ni K., Zhu L., Zhang Q., Yin M., Zhang Z., Wei W., Preece D. A., Li B. M. (2024). Chinese version of the Perth Alexithymia Questionnaire: Psychometric properties and clinical applications. General Psychiatry.

[B12-ejihpe-16-00030] Connelly M., Denney D. R. (2007). Regulation of emotions during experimental stress in alexithymia. Journal of Psychosomatic Research.

[B13-ejihpe-16-00030] Edwards E. R. (2022). Posttraumatic stress and alexithymia: A meta-analysis of presentation and severity. Psychological Trauma.

[B14-ejihpe-16-00030] Fang S., Chung M. C., Wang Y. (2020). The impact of past trauma on psychological distress: The roles of defence mechanisms and alexithymia. Frontiers in Psychology.

[B15-ejihpe-16-00030] Finlay-Jones A. L., Rees C. S., Kane R. T. (2015). Self-compassion, emotion regulation and stress among Australian psychologists: Testing an emotion regulation model of self-compassion using structural equation modeling. PLoS ONE.

[B16-ejihpe-16-00030] Fritz M. S., MacKinnon D. P. (2007). Required sample size to detect the mediated effect. Psychological Science.

[B17-ejihpe-16-00030] Gilbert P. (2014). The origins and nature of compassion focused therapy. British Journal of Clinical Psychology.

[B18-ejihpe-16-00030] Gratz K. L., Roemer L. (2004). Multidimensional assessment of emotion regulation and dysregulation: Development, factor structure, and initial validation of the difficulties in emotion regulation scale. Journal of Psychopathology and Behavioral Assessment.

[B19-ejihpe-16-00030] Gross J. J. (2015). Emotional regulation: Current status and future prospects. Psychological Inquiry.

[B20-ejihpe-16-00030] Hallion L. S., Steinman S. A., Tolin D. F., Diefenbach G. J. (2018). Psychometric properties of the difficulties in emotion regulation scale (DERS) and its short forms in adults with emotional disorders. Frontiers in Psychology.

[B21-ejihpe-16-00030] Hayes A. F. (2022). Introduction to mediation, moderation, and conditional process analysis: A regression-based approach.

[B22-ejihpe-16-00030] Hetzel-Riggin M. D., Meads C. L. (2016). Interrelationships among three avoidant coping styles and their relationship to trauma, peritraumatic distress, and posttraumatic stress disorder. The Journal of Nervous and Mental Disease.

[B23-ejihpe-16-00030] Hoeboer C. M., Karaban I., Karchoud J. F., Olff M., van Zuiden M. (2024). Validation of the PCL-5 in Dutch trauma-exposed adults. BMC Psychology.

[B24-ejihpe-16-00030] Ibrahim H., Ertl V., Catani C., Ismail A. A., Neuner F. (2018). The validity of posttraumatic stress disorder checklist for DSM-5 (PCL-5) as screening instrument with Kurdish and Arab displaced populations living in the Kurdistan region of Iraq. BMC Psychiatry.

[B25-ejihpe-16-00030] Inwood E., Ferrari M. (2018). Mechanisms of change in the relationship between self-compassion, emotion regulation, and mental health: A systematic review. Applied Psychology: Health and Well-Being.

[B26-ejihpe-16-00030] Kilpatrick D. G., Resnick H. S., Milanak M. E., Miller M. W., Keyes K. M., Friedman M. J. (2013). National estimates of exposure to traumatic events and PTSD prevalence using DSM-IV and DSM-5 criteria. Journal of Traumatic Stress.

[B27-ejihpe-16-00030] Kindred R., Nedeljkovic M., Bates G., Collins F. (2024). Understanding emotion regulation in PTSD and complex PTSD. Understanding emotional regulation: New research.

[B28-ejihpe-16-00030] Kooiman C. G., Spinhoven P., Trijsburg R. W. (2002). The assessment of alexithymia: A critical review of the literature and a psychometric study of the Toronto Alexithymia Scale-20. Journal of Psychosomatic Research.

[B29-ejihpe-16-00030] Larionow P., Mudlo-Glagolska K., Preece D. A. (2025). Is alexithymia a trait or a state? Temporal stability in a three-wave longitudinal study. Journal of Clinical Medicine.

[B30-ejihpe-16-00030] Lashkari A., Dehghani M., Sadeghi-Firoozabadi V., Heidari M., Khatibi A. (2021). Further support for the psychometric properties of the Farsi version of Perth alexithymia questionnaire. Frontiers in Psychology.

[B31-ejihpe-16-00030] Lilly M. M., Valdez C. E. (2012). The unique relationship of emotion regulation and alexithymia in predicting somatization versus PTSD symptoms. Journal of Aggression, Maltreatment & Trauma.

[B32-ejihpe-16-00030] Litz B. T., Litz B. T., Gray M. J. (2002). Emotional numbing in posttraumatic stress disorder: Current and future research directions. Australian & New Zealand Journal of Psychiatry.

[B33-ejihpe-16-00030] Luminet O., Nielson K. A. (2025). Alexithymia: Toward an experimental processual affective science with effective interventions. Annual Review of Psychology.

[B34-ejihpe-16-00030] Lyvers M., Randhawa A., Thorberg F. A. (2020). Self-compassion in relation to alexithymia, empathy, and negative mood in young adults. Mindfulness.

[B35-ejihpe-16-00030] MacBeth A., Gumley A. (2012). Exploring compassion: A meta-analysis of the association between self-compassion and psychopathology. Clinical Psychology Review.

[B36-ejihpe-16-00030] McBride N. L., Bates G. W., Elphinstone B., Whitehead R. (2022). Self-compassion and social anxiety: The mediating effect of emotion regulation strategies and the influence of depressed mood. Psychology and Psychotherapy: Theory, Research and Practice.

[B37-ejihpe-16-00030] Mehta A., Moeck E., Preece D. A., Koval P., Gross J. J. (2025). Alexithymia and emotion regulation: The role of emotion intensity. Affective Science.

[B38-ejihpe-16-00030] Miethe S., Wigger J., Wartemann A., Fuchs F. O., Trautmann S. (2023). Posttraumatic stress symptoms and its association with rumination, thought suppression and experiential avoidance: A systematic review and meta-analysis. Journal of Psychopathology and Behavioral Assessment.

[B39-ejihpe-16-00030] Neff K. D. (2003). The development and validation of a scale to measure self-compassion. Self and Identity.

[B40-ejihpe-16-00030] Neff K. D. (2023). Self-compassion: Theory, method, research, and intervention. Annual Review of Psychology.

[B41-ejihpe-16-00030] Neff K. D., Long P., Knox M. C., Davidson O., Kuchar A., Costigan A., Williamson Z., Rohleder N., Toth-Kiraly I., Breines J. G. (2018). The forest and the trees: Examining the association of self-compassion and its positive and negative components with psychological functioning. Self and Identity.

[B42-ejihpe-16-00030] Oglodek E. A. (2022). Alexithymia and emotional deficits related to posttraumatic stress disorder: An investigation of content and process disturbances. Case Reports in Psychiatry.

[B43-ejihpe-16-00030] Ozer E. J., Weiss D. S. (2004). Who develops posttraumatic stress disorder?. Current Directions in Psychological Science.

[B44-ejihpe-16-00030] Panayiotou G., Leonidou C., Constantinou E., Michaelides M. P. (2020). Self-awareness in alexithymia and associations with social anxiety. Current Psychology.

[B45-ejihpe-16-00030] Podsakoff P. M., Mackenzie S. B., Podsakoff N. P., Jeong-Yeon L. (2003). Common method biases in behavioral research. A critical review of the literature and recommended remedies. Journal of Applied Psychology.

[B46-ejihpe-16-00030] Preece D. A., Becerra R., Robinson K., Dandy J., Allan A. (2018). The psychometric assessment of alexithymia: Development and validation of the Perth alexithymia questionnaire. Personality and Individual Differences.

[B47-ejihpe-16-00030] Preece D. A., Mehta A., Petrova K., Sikka P., Bjureberg J., Becerra R., Gross J. (2023). Alexithymia and emotion regulation. Journal of Affective Disorders.

[B48-ejihpe-16-00030] Putica A. (2024). Examining the role of emotion and alexithymia in cognitive behavioural therapy outcomes for posttraumatic stress disorder: Clinical implications. The Cognitive Behaviour Therapist.

[B49-ejihpe-16-00030] Putica A., O’Donnell M. L., Felmingham K. L., Van Dam N. T. (2023). Emotion response disconcordance among trauma-exposed adults: The impact of alexithymia. Psychological Medicine.

[B50-ejihpe-16-00030] Putica A., Van Dam N. T., Felmingham K., Lawrence-Wood E., McFarlane A., O’Donnell M. (2024). Interactive relationship between alexithymia, psychological distress and posttraumatic stress disorder symptomology across time. Cognition and Emotion.

[B51-ejihpe-16-00030] Putica A., Van Dam N. T., Steward T., Agathos J., Felmingham K., O’Donnell M. (2021). Alexithymia in post-traumatic stress disorder is not just emotion numbing: Systematic review of neural evidence and clinical implications. Journal of Affective Disorders.

[B52-ejihpe-16-00030] Scheppes G., Scheibe S., Suri G., Radu P., Blechert J., Gross J. J. (2014). Emotion regulation choice: A conceptual framework and supporting evidence. Journal of Experimental Psychology: General.

[B53-ejihpe-16-00030] Seligowski A. V., Miron L. R., Ocutt H. K. (2015). Relations among self-compassion, PTSD symptoms and psychological health in a trauma-exposed sample. Mindfulness.

[B54-ejihpe-16-00030] Tabachnick B. G., Fidell L. S. (2019). Using multivariate statistics.

[B55-ejihpe-16-00030] Taylor G. J., Porcelli P., Bagby M. (2024). Alexithymia: A defence of the original conceptualization of the construct and a critique of the attention-appraisal model. Clinical Neuropsychiatry.

[B56-ejihpe-16-00030] Tull M. T., Vidaña A. G., Betts J. E., Tull M. T., Kimbrel N. A. (2020). Emotion regulation difficulties in PTSD. Emotion in posttraumatic stress disorder: Etiology, assessment, neurobiology, and treatment.

[B57-ejihpe-16-00030] Weathers F. W., Litz B. T., Keane T. M., Palmieri P. A., Marx B. P., Schnurr P. P. (2013). The PTSD checklist for DSM-5 (PCL-5).

[B58-ejihpe-16-00030] Weiss N. H., Darosh A. G., Contractor A. A., Schick M. M., Dixon-Gordon K. L. (2019). Confirmatory validation of the factor structure and psychometric properties of the difficulties in emotion regulation scale-positive. Journal of Clinical Psychology.

[B59-ejihpe-16-00030] Weiss N. H., Dixon-Gordon K. L., Peasant C., Sullivan T. P. (2018). An examination of the role of difficulties regulating positive emotions in posttraumatic stress disorder. Journal of Traumatic Stress.

[B60-ejihpe-16-00030] Weiss N. H., Forkus S. R., Contractor A. A., Dixon-Gordon K. L. (2020). The interplay of negative and positive emotion dysregulation on mental health outcomes among trauma exposed community individuals. Psychological Trauma: Theory, Research and Policy.

[B61-ejihpe-16-00030] Weiss N. H., Gratz K. L., Lavender J. M. (2015). Factor structure and initial validation of a multidimensional measure of difficulties in the regulation of positive emotions: The DERS-Positive. Behavior Modification.

[B62-ejihpe-16-00030] Winders S. J., Murphy O., Looney K., O’Reilly G. (2022). Self-compassion, trauma, and posttraumatic stress disorder: A systematic review. Clinical Psychology & Psychotherapy.

[B63-ejihpe-16-00030] Zeller M., Yuval K., Nitzan-Assayag Y., Bernstein A. (2015). Self-compassion in recovery following potentially traumatic stress: Longitudinal study of at-risk youth. Journal of Abnormal Child Psychology.

