# Supplementary material for: Alexithymia and Symptoms of Post-Traumatic Stress Disorder: The Mediation Roles of Self-Compassion and Deficits in Emotion Regulation
Source: Eur J Investig Health Psychol Educ. 2026 Feb 18;16(2):30. doi: 10.3390/ejihpe16020030 (PMC12940040; doi:10.3390/ejihpe16020030)
Supplement: Supplementary file 1 [file ejihpe-16-00030-s001.zip › ejihpe-3965611-supplementary.pdf]

Table S1. *Total, Indirect and Direct Effects of Serial Mediation: Alexithymia and Total PTSD Symptoms With Self-Compassion (SCS), Difficulties regulating Negative emotions (DERS-N and Difficulties Regulating Positive Emotions (DERS-P) as Mediators*

	B	*SE*	*p*	LL	UL	ES
Total effect (of Alexithymia on PTSD)	.358	.028	< .0001	.000	0.42	.342
Indirect effect through SCS	.019	.018		-.015	.056	.031
Indirect effect through DERS-N	.106	.022		.067	.151	.173
Indirect effect through DERS-P	.030	.013		.006	.059	.049
Indirect effect through SCS and DERS-N	.057	.012		.036	.083	.937
Indirect effect through SCS and DERS-P	-.003	.002		-.001	.001	-001
Direct effect (*c'*)	.148	.033	< .0001	.083	.214	.242

Note> *B*, unstandardised coefficient. *SE*, bootstrap Standard Error. LL, bootstrap confidence interval Lower Limit. UL, bootstrap confidence interval Upper Limit. ES, standardised Effect Size.

Table S2. *Total, Indirect and Direct Effects of Serial Mediation: Alexithymia and Re-experiencing PTSD Symptoms (ReX) With Self-Compassion (SCS), Difficulties regulating Negative emotions (DERS-N and Difficulties Regulating Positive Emotions (DERS-P) as Mediators*

	B	*SE*	*p*	LL	UL	ES
Total effect (of Alexithymia on ReX)	.073	.008	< .0001	.000	0.57	.440
Indirect effect through SCS	.000	.005		-.010	.011	.002
Indirect effect through DERS-N	.025	.006		.014	.038	.153
Indirect effect through DERS-P	-.004	.004		-.040	.012	.023
Indirect effect through SCS and DERS-N	.014	.004		.008	.021	.125
Indirect effect through SCS and DERS-P	-.000	.001		-.002	.001	-002
Direct effect (*c'*)	.031	.011	< .01	.010	.051	.183

Note> *B*, unstandardised coefficient. *SE*, bootstrap Standard Error. LL, bootstrap confidence interval Lower Limit. UL, bootstrap confidence interval Upper Limit. ES, standardised Effect Size.

Table S3. *Total, Indirect and Direct Effects of Serial Mediation: Alexithymia and Avoidance PTSD Symptoms With Self-Compassion (SCS), Difficulties regulating Negative emotions (DERS-N and Difficulties Regulating Positive Emotions (DERS-P) as Mediators*

	B	*SE*	*p*	LL	UL	ES
Total effect (of Alexithymia on Avoidance)	.032	.004	$< .0001$	.000	0.24	.394
Indirect effect through SCS	-.004	.003		-.010	.001	.018
Indirect effect through DERS-N	.017	.003		.011	.024	.290
Indirect effect through DERS-P	-.001	.002		-.005	.003	.034
Indirect effect through SCS and DERS-N	.009	.002		.006	.013	.113
Indirect effect through SCS and DERS-P	-.003	.002		-.000	.001	010
Direct effect (*c'*)	.011	.005	$< .05$	.038	.001	.009

Note> *B*, unstandardised coefficient. *SE*, bootstrap Standard Error. LL, bootstrap confidence interval Lower Limit. UL, bootstrap confidence interval Upper Limit. ES, standardised Effect Size.

Table S4. *Total, Indirect and Direct Effects of Serial Mediation: Alexithymia and Negative Alteration in mood and Cognition (NAMC) Symptoms With Self-Compassion (SCS), Difficulties Regulating Negative emotions (DERS-N and Difficulties Regulating Positive Emotions (DERS-P) as Mediators*

	B	*SE*	*p*	LL	UL	ES
Total effect (of Alexithymia on NAMC)	.141	.011	< .0001	.000	0.12	.340
Indirect effect through SCS	.082	.010		.068	.102	.058
Indirect effect through DERS-N	.037	.008		.021	.053	.153
Indirect effect through DERS-P	.013	.006		.002	.024	.053
Indirect effect through SCS and DERS-N	.020	.005		.011	.030	.082
Indirect effect through SCS and DERS-P	-.001	.001		-.004	.000	-006
Direct effect (*c'*)	.141	.011	< .0001	.000	.119	.162

Note> *B*, unstandardised coefficient. *SE*, bootstrap Standard Error. LL, bootstrap confidence interval Lower Limit. UL, bootstrap confidence interval Upper Limit. ES, standardised Effect Size.

Table S5. *Total, Indirect and Direct Effects of Serial Mediation: Alexithymia and Altered Arousal and Reactivity(AAR) Symptoms With Self-Compassion (SCS), Difficulties regulating Negative Emotions (DERS-N and Difficulties Regulating Positive Emotions (DERS-P) as Mediators*

	B	*SE*	*p*	LL	UL	ES
Total effect (of Alexithymia on AAR)	.048	.011	< .0001	.000	0.26	.070
Indirect effect through SCS	.009	.006		-.002	.021	.120
Indirect effect through DERS-N	.027	.007		.014	.042	.139
Indirect effect through DERS-P	.015	.005		.006	.025	.076
Indirect effect through SCS and DERS-N	.015	.004		.008	.023	.075
Indirect effect through SCS and DERS-P	-.002	.001		-.004	.000	-008
Direct effect (*c'*)	.048	.011	< .0001	.000	.026	.070

Note> *B*, unstandardised coefficient. *SE*, bootstrap Standard Error. LL, bootstrap confidence interval Lower Limit. UL, bootstrap confidence interval Upper Limit. ES, standardised Effect Size.
